# Chemotherapy-associated coronary thrombosis presenting as acute coronary syndrome in breast cancer: a case report

**DOI:** 10.1186/s12872-026-05512-6

**Published:** 2026-01-09

**Authors:** Oya Imadoglu, Emre Emrah Demirci, Sefa Sural

**Affiliations:** 1 Mersin City Training and Research Hospital, Korukent Mah. 96015 Sok. Mersin Entegre Sağlık Kampüsü, Toroslar, Mersin, 33240 Turkey; 2 Vocational School of Health Service, Toros University, Bahçelievler 1835 sokak. No: 4 Yenişehir, Mersin, 33140 Turkey

**Keywords:** Acute coronary syndrome, Coronary thrombosis, Breast cancer, Doxorubicin, Cyclophosphamide, Cardio-oncology

## Abstract

**Background:**

Cancer patients face a high risk of thromboembolic complications due to disease-, treatment-, and patient-related factors. Chemotherapy regimens such as anthracyclines and alkylating agents can increase both venous and arterial thrombosis, leading to significant morbidity.

**Case summary:**

A 44-year-old woman with invasive ductal carcinoma of the right breast underwent right mastectomy and received adjuvant chemotherapy with doxorubicin (60 mg/m²) and cyclophosphamide (600 mg/m²). Five days after her second treatment cycle, she presented with chest pain. Electrocardiography showed inferolateral ST-segment elevation, and echocardiography revealed a left ventricular ejection fraction (LVEF) of 38%. Coronary angiography demonstrated total occlusion of the distal left anterior descending and circumflex arteries. She was treated with dual antiplatelet therapy, low-molecular-weight heparin, and a glycoprotein IIb/IIIa inhibitor. Follow-up angiography on day five showed complete thrombus resolution and restoration of TIMI III flow. During long-term follow-up, her left ventricular function recovered (LVEF 50%) and her cancer remained in complete remission.

**Conclusion:**

This case illustrates a rare but clinically important complication of adjuvant doxorubicin–cyclophosphamide therapy, presenting as subacute inferolateral myocardial infarction secondary to coronary thrombosis. Awareness of this potential adverse effect and early management are crucial for improved outcomes.

**Supplementary Information:**

The online version contains supplementary material available at 10.1186/s12872-026-05512-6.

## Introduction

Cancer patients have a well-documented predisposition to both venous and arterial thromboembolic events, which constitute a major source of morbidity and mortality [1]. This heightened risk arises from the interplay of malignancy-associated hypercoagulability, patient-specific factors, and the prothrombotic effects of anticancer therapies [2]. Chemotherapeutic agents, in particular, can induce endothelial injury and platelet activation, thereby promoting the development of venous as well as arterial thrombosis [3]. While venous thromboembolism is more frequently recognized, arterial thrombotic events—including acute coronary syndromes (ACS)—are less common but carry significant clinical implications [4].

Among chemotherapeutic drugs, doxorubicin and cyclophosphamide are widely used in breast cancer treatment and are both associated with well-known cardiotoxicity [5]. Although their cardiotoxicity typically manifests as left ventricular dysfunction or heart failure, chemotherapy-induced coronary thrombosis leading to myocardial infarction remains exceedingly rare [4, 6–8].

Here, we describe a case of subacute inferolateral myocardial infarction secondary to coronary thrombosis occurring after adjuvant doxorubicin–cyclophosphamide therapy in a patient with breast cancer. This report underscores the importance of maintaining clinical vigilance and promptly recognizing this uncommon yet potentially life-threatening complication.

## Case presentation

A 44-year-old woman with a history of malignancy presented to the emergency department five days after her second course of chemotherapy, with a two-day history of intermittent, pressure-like chest pain. The pain was retrosternal, radiating to the left arm, and associated with mild dyspnea; neither nausea nor diaphoresis was present.

**Fig. 1:**
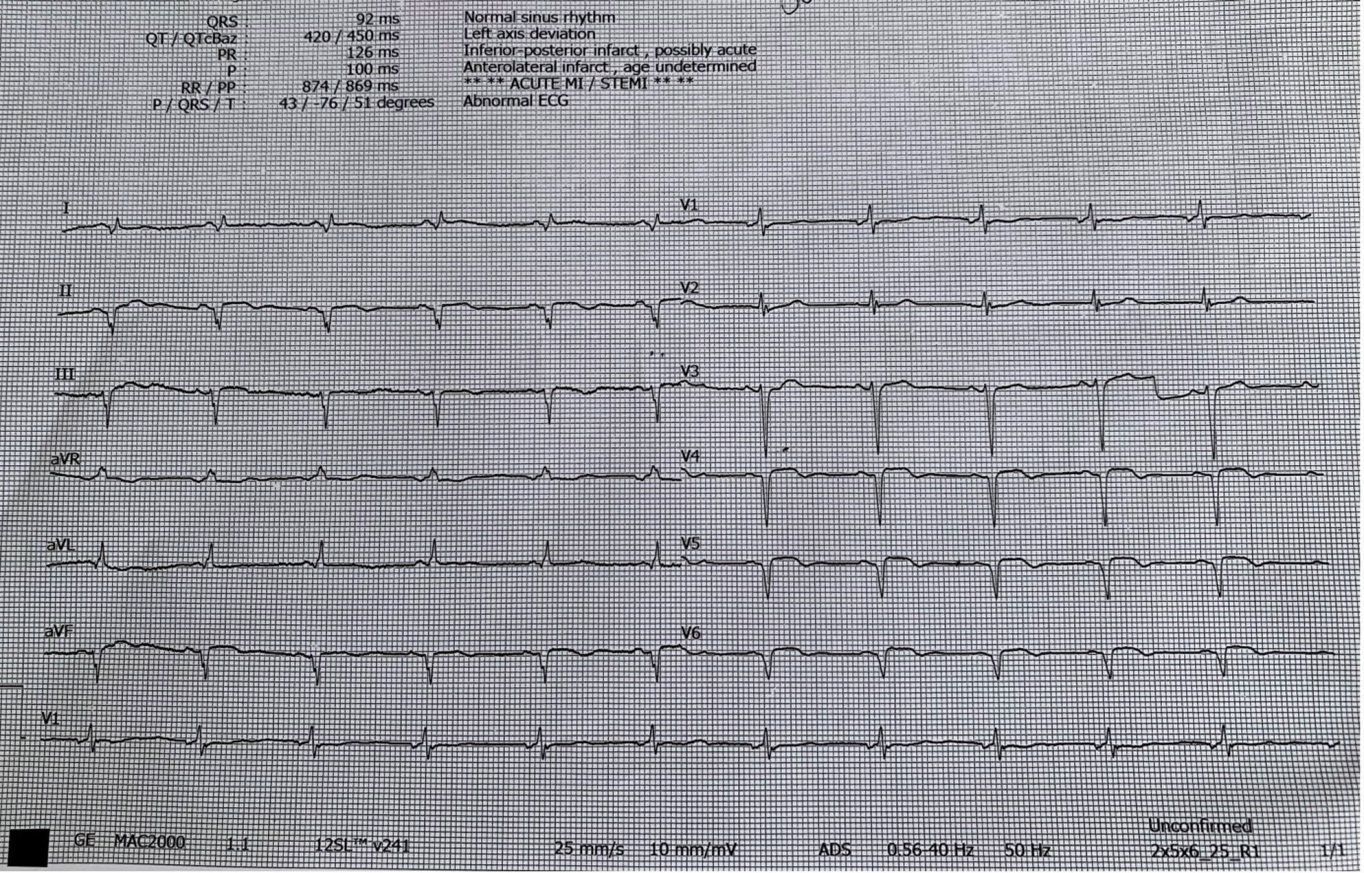
Patient’s electrocardiogram showing pathological Q waves in leads II, III, aVF, and V4–V6, with ST-segment elevation at presentation to the emergency department

On physical examination, postoperative scar tissue and mild right arm edema were noted. Cardiac auscultation revealed a grade 1–2/6 systolic murmur at the apex. There were no cutaneous lesions or peripheral findings suggestive of disseminated thrombosis. Electrocardiography demonstrated sinus rhythm with inferolateral pathological Q waves in leads II, III, aVF, and V4–V6, as well as 1 mm ST-segment elevation (Fig. 1). Based on ECG findings demonstrating ST-segment elevation, the patient was diagnosed with subacute STEMI and admitted to the coronary intensive care unit. Transthoracic echocardiography demonstrated a left ventricular ejection fraction (LVEF) of 38%, hypokinesis of the anterior and inferolateral walls, and mild mitral regurgitation, with no evidence of intracardiac thrombus. Laboratory testing revealed an elevated high-sensitivity troponin I (hs-Troponin I) level of 877.0 ng/L (reference range: 0–47 ng/L), a platelet count of 335 × 10³/µL, hemoglobin level of 11.4 g/dL, and normal renal function. Thoracic computed tomography performed at admission showed no findings of viral pneumonia, and a SARS-CoV-2 PCR test was negative.

Her medical history revealed that approximately three months before the myocardial infarction, she had been diagnosed with invasive ductal carcinoma of the right breast. Immunohistochemical analysis showed estrogen and progesterone receptor positivity, HER2 negativity, and a Ki-67 proliferation index of 25–30%. Pathological staging was T2N2bM0, corresponding to Stage III disease. PET-CT demonstrated no distant metastases. The pathology report described intramammary lymph node metastasis, referring to metastatic involvement of lymph nodes located within the breast parenchyma rather than within the axillary basin. Her Eastern Cooperative Oncology Group (ECOG) performance status was 0.

She subsequently underwent a right mastectomy with excision of an intramammary metastatic lymph node. She had no known comorbidities or regular medication use, reported smoking one pack of cigarettes per day, and had no personal or family history of coronary artery disease. Although smoking is an established cardiovascular risk factor, her pre-chemotherapy cardiovascular evaluation revealed no evidence of underlying atherosclerosis: ECG showed no ischemic changes, echocardiography demonstrated normal LV function (LVEF 60%), and the coronary artery calcium score on PET-CT was zero. According to the 2024 ESC RF-CL (Risk Factor-weighted Clinical Likelihood) model [9], her pretest probability of obstructive coronary disease was approximately 1%, placing her in the very-low-risk category. Adjuvant chemotherapy with doxorubicin (60 mg/m²) and cyclophosphamide (600 mg/m²) had been initiated.

**Fig. 2:**
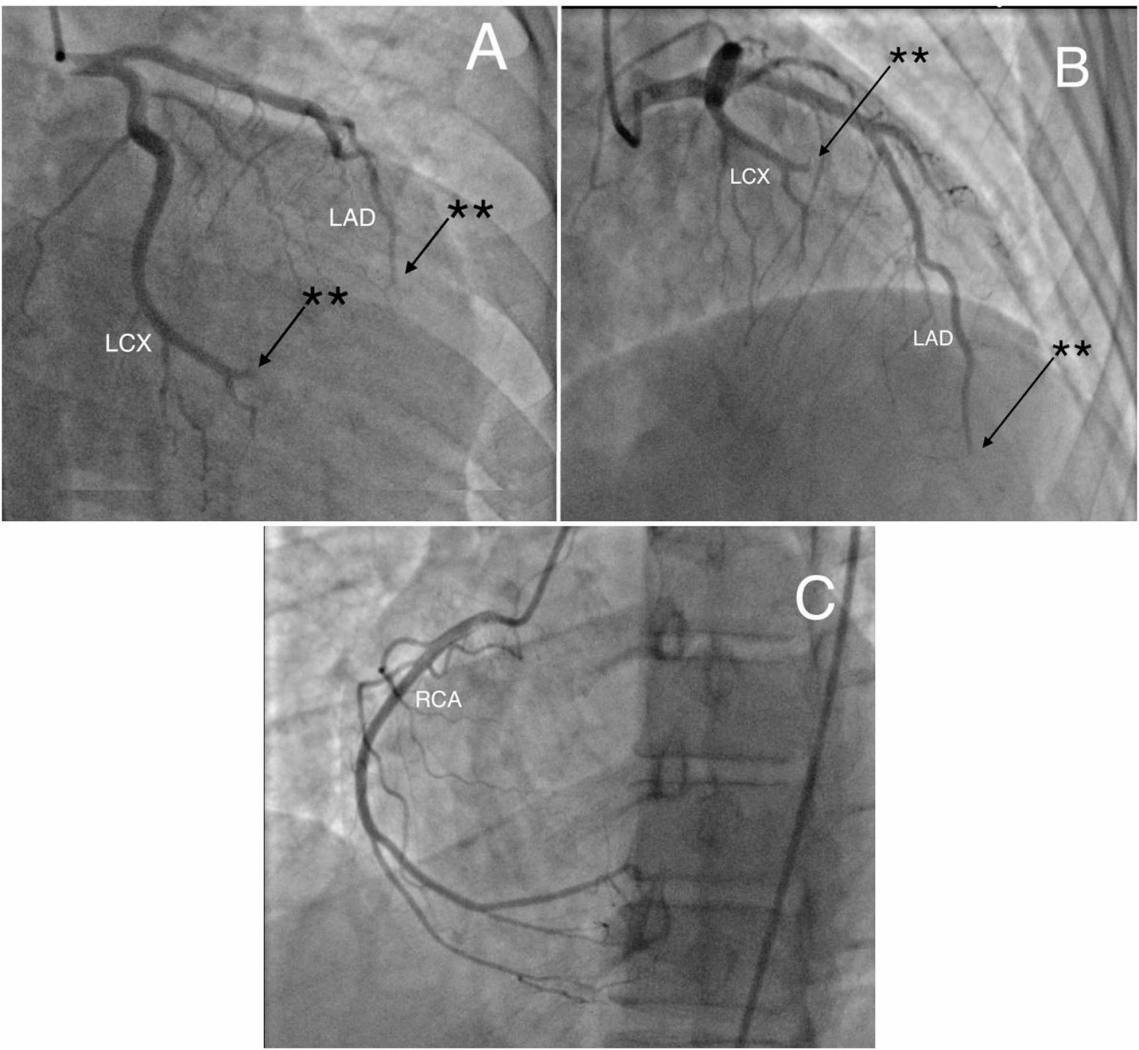
Initial coronary angiography performed after admission demonstrating total occlusion of both arteries **(**asterisks indicate the occluded segments). **A** Right anterior oblique (RAO) caudal projection; (**B**) Left anterior oblique (LAO) cranial projection; (**C**) angiographic image demonstrating a patent right coronary artery. *LAD* Left anterior descending artery, *LCX* Left circumflex artery, *RCA* Right coronary artery

Coronary angiography (CAG) revealed total occlusion of the distal left anterior descending (LAD) and circumflex (LCX) arteries, with a normal right coronary artery (RCA) (Fig. 2 and Supplementary Video 1). Balloon angioplasty was performed on the distal LAD and LCX lesions; however, no restoration of coronary perfusion was achieved, and flow remained TIMI 0, indicating persistent total occlusion. On the first day of treatment, dual antiplatelet therapy (DAPT), a parenteral anticoagulant, and an intravenous glycoprotein IIb/IIIa inhibitor (tirofiban; administered as a 25 µg/kg intravenous loading dose infused over 5 min, followed by a continuous infusion of 0.15 µg/kg/min for a total duration of 18 h) were initiated. After completion of the tirofiban infusion, antithrombotic therapy was continued with DAPT and low-molecular-weight heparin for an additional four days.

**Fig. 3:**
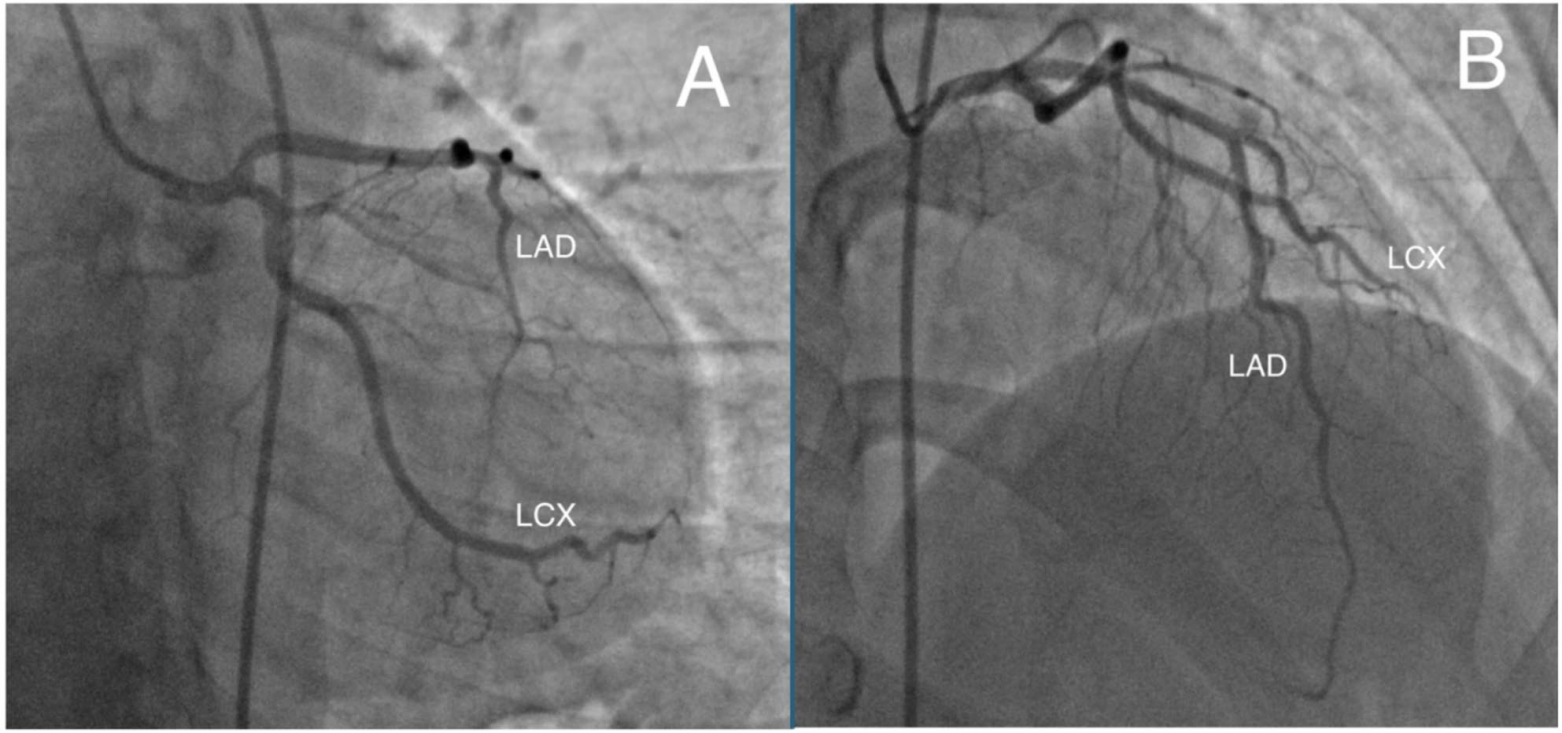
Follow-up coronary angiography on day 5 demonstrating recanalization of previously occluded arteries. **A** Right anterior oblique (RAO) caudal projection; (**B**) Left anterior oblique (LAO) cranial projection. *LAD* Left anterior descending artery, *LCX* Left circumflex artery

Follow-up CAG on day five showed complete thrombus resolution and restoration of TIMI III flow in both the LAD and LCX arteries (Fig. 3 and Supplementary Video 2). Laboratory evaluation revealed normal fibrinogen, D-dimer, and homocysteine levels. Genetic testing for hereditary thrombophilia showed no antithrombin III or Factor V Leiden mutations. Protein C activity was normal, whereas protein S activity was mildly decreased.

Throughout hospitalization, the patient remained hemodynamically stable and asymptomatic. She was observed for one additional day following the control angiography and discharged on hospital day six. The oncology team was informed, and multidisciplinary follow-up was recommended.

Following the myocardial infarction, doxorubicin and cyclophosphamide therapy were discontinued, and treatment was continued with paclitaxel. Given the estrogen and progesterone receptor positivity, adjuvant endocrine therapy with letrozole 2.5 mg daily was initiated and remains ongoing. Approximately twelve months after the MI, the patient achieved complete remission. At her 44-month follow-up, she remained in complete oncologic remission but continued to smoke one pack of cigarettes per day. During follow-up, she was repeatedly advised to quit smoking; however, she chose to continue. Transthoracic echocardiography at that time demonstrated near-complete recovery of systolic function, with an ejection fraction of 50%. The patient is currently maintained on acetylsalicylic acid 100 mg daily, ramipril 2.5 mg, and carvedilol 6.25 mg.

## Discussion

Cardiovascular complications, particularly thromboembolic events, represent important adverse effects of cancer therapy [10, 11]. The classical mechanisms of thrombosis described in Virchow’s triad—endothelial injury, stasis, and hypercoagulability—are often exacerbated during chemotherapy [12].

The etiology of venous or arterial thrombosis in cancer patients may stem from both the procoagulant processes induced by the malignancy itself and the thrombogenic effects of chemotherapeutic agents [4]. Proposed mechanisms include the release of procoagulant factors and proinflammatory cytokines from cancer cells, increased markers of platelet activation, coronary embolization of tumor cells [11, 13–16], endothelial dysfunction [17], cardiac tumors or metastases [18, 19], coronary artery compression by thoracic malignancies [20], chemotherapy-induced vascular injury, coronary effects such as vasospasm, endothelial dysfunction, spontaneous thrombosis, accelerated atherosclerosis [11, 14, 17, 21], and radiation-induced vasculitis [22].

In this patient, the exact cause of thrombosis—whether due to the malignancy itself or chemotherapy—could not be definitively determined. However, the incidence of chemotherapy-associated arterial thrombosis in breast cancer has been reported to be low, ranging from 1% to 4.8% [23–25]. Both anthracyclines and alkylating agents are known to cause vascular injury and platelet activation through endothelial dysfunction and oxidative stress [3]. Doxorubicin can directly bind to endothelial nitric oxide synthase (eNOS), leading to reduced nitric oxide (NO) production and consequent superoxide formation. The resulting decrease in NO levels promotes a procoagulant endothelial phenotype and impairs vasodilation [26]. Cyclophosphamide, in particular, may lead to direct endothelial injury resulting from the extravasation of toxic metabolites, followed by cardiomyocyte damage, interstitial hemorrhage, and edema formation. Moreover, the development of microemboli may contribute to ischemic myocardial injury [5, 27–29].

Arterial thrombosis following doxorubicin–cyclophosphamide therapy has been reported only rarely. For instance, Jang et al. described a patient receiving adjuvant doxorubicin–cyclophosphamide chemotherapy for invasive ductal breast carcinoma who developed acute arterial thrombosis of the left brachial, radial, and ulnar arteries five days after the second cycle [30]. Similarly, our patient developed acute coronary syndrome five days after the second cycle of the same regimen. This temporal association between chemotherapy administration and the thrombotic event supports a possible causal relationship between the doxorubicin–cyclophosphamide combination and arterial thrombosis. These mechanisms may explain the clinical presentation and angiographic findings in our patient, who developed coronary thrombosis in the absence of preexisting atherosclerotic disease.

While the patient’s pre-chemotherapy evaluation indicated a very low likelihood of obstructive coronary disease, her chronic smoking may still have acted as a synergistic pro-thrombotic factor in the setting of chemotherapy-related endothelial injury.

Although the management of acute coronary syndrome (ACS) in cancer patients generally follows standard clinical guidelines [31], antiplatelet therapy and PCI can be limited by treatment-related thrombocytopenia [32, 33]. In our patient, simultaneous total occlusion of the LAD and LCX arteries in the absence of thrombocytopenia suggested fragmented thrombus embolization. Significant clinical improvement was achieved with tirofiban, heparin, and dual antiplatelet therapy, and the patient was discharged on DAPT and low-molecular-weight heparin. This case underscores the importance of tailoring antithrombotic strategies in the management of ACS among patients with cancer [34]. Simultaneous thrombotic occlusion of the distal LAD and LCX is an extremely rare clinical condition. In similar cases reported in the literature, dual-vessel involvement is often attributed to local in-situ thrombosis developing in a prothrombotic environment, and the exact source of a possible embolus frequently remains undetermined [35, 36]. In our patient, chemotherapy-induced endothelial injury and hypercoagulability may have contributed to local thrombus formation with subsequent distal fragmentation. Also, although not shown on angiography or echocardiography, the possibility of a proximally formed thrombus that fragmented and embolized into the distal branches cannot be ruled out; however, this remains a clinical hypothesis and cannot be confirmed with the available data. Therefore, the exact origin of the thrombus in this case cannot be identified, and the mechanisms described should be seen as plausible explanations rather than definitive interpretations.

In conclusion, this case illustrates that coronary thrombosis caused by chemotherapy is a rare but clinically important occurrence following adjuvant doxorubicin–cyclophosphamide treatment. Clinicians should maintain a high index of suspicion for arterial thrombotic complications when cancer patients present with chest pain during or shortly after chemotherapy. Prompt recognition and early initiation of antithrombotic therapy are crucial for improving patient outcomes. Further studies and case collections are needed to elucidate the mechanisms and optimal management of chemotherapy-related coronary thrombosis.

## Supplementary Information

Supplementary Material 1.

Supplementary Material 2.

## Data Availability

All data generated or analyzed in this study are available in the published article. For any additional questions, please contact the corresponding author.

## Abbreviations

ACS: Acute coronary syndrome
CAG: Coronary angiography
DAPT: Dual antiplatelet therapy
ECOG: Eastern Cooperative Oncology Group
EF: Ejection fraction
LAD: Left anterior descending
LCX: Left circumflex artery
LVEF: Left ventricular ejection fraction
PET-CT: Positron Emission Tomography – Computed Tomography
PCI: Percutaneous coronary intervention
MI: Myocardial infarction
STEMI: ST-segment elevation myocardial infarction
